# Exploring the Neurological Manifestations of Leprosy: Clinical Insights and Implications

**DOI:** 10.7759/cureus.77799

**Published:** 2025-01-21

**Authors:** Masoumeh Rashidi, Jamir Pitton Rissardo, Vishnu V Byroju, Ana Leticia Fornari Caprara, Fatemeh Rashidi, Omesh Prathiraja, Hania Moharam, Christopher C Elendu, Mallak Bahar, Maleesha Jayasinghe

**Affiliations:** 1 Medical Education, Nanjing Medical University, Nanjing, CHN; 2 Neurology, Cooper University Hospital, Camden, USA; 3 Neurology, Cooper Medical School of Rowan University, Camden, USA; 4 Neurology, Universidade Federal de Santa Maria, Santa Maria, BRA; 5 Medicine, Nanjing Medical University, Nanjing, CHN; 6 Medicine and Surgery, Nanjing Medical University, Nanjing, CHN

**Keywords:** cutaneous manifestations of leprosy, hansen's disease, leprosy, leprosy complications, leprosy transmission, leprosy treatment, manifestations of leprosy, mycobacterium leprae

## Abstract

Leprosy is one of the neglected tropical diseases, and its elimination remains a public health problem globally. This manuscript comprehensively explores the neurological manifestations of leprosy, offering clinical insights and implications for the diagnosis, management, and understanding of this disease. Beginning with a review of historical context, etiology, and epidemiology, we delve into the pathophysiology of leprosy neuropathy, highlighting mechanisms of nerve damage and immune response. Peripheral nerve involvement, including sensory and motor deficits, nerve enlargement, and deformities, are discussed in detail, along with the challenges in diagnosis and management. Psychological and social implications of neurological deficits in leprosy are addressed, emphasizing the importance of holistic care and support. Emerging trends in neuroimaging and molecular diagnostics offer promising avenues for improved diagnosis and therapeutic interventions. Novel therapeutic strategies are identified to enhance treatment efficacy and prevent disability in leprosy neuropathy by targeting immunomodulatory pathways, antibacterial agents, and a personalized medicine approach.

## Introduction and background

Leprosy, also known as Hansen’s disease, holds a unique place in human history, dating back thousands of years. Ancient texts and archaeological findings indicate that leprosy has afflicted civilizations across the globe, with mentions found in Egyptian, Indian, Chinese, and Hebrew writings [1]. Throughout history, leprosy has been shrouded in stigma and fear, leading to the segregation of affected individuals in many societies. However, advancements in medical understanding and social attitudes have gradually transformed perceptions of leprosy [2]. The etiological agent of leprosy is *Mycobacterium leprae (M. leprae)*, an acid‐fast bacillus (AFB) first identified by Norwegian scientist Gerhard Armauer Hansen in 1873 [3]. *M. leprae* primarily targets the skin and peripheral nerves, leading to the characteristic skin lesions and neurological manifestations associated with the disease. This study aims to narratively review the neurological manifestations of leprosy, its epidemiology, pathophysiology, classification, and management.

Epidemiology

Leprosy remains a public health challenge in various parts of the world. Despite significant difficulties in disease control and elimination efforts, leprosy remains endemic in several regions, with ongoing transmission and new cases reported annually. In this context, the global prevalence of leprosy has decreased substantially over the past few decades. According to the World Health Organization (WHO), the prevalence rate of leprosy dropped from approximately 5.2 million cases in the 1980s to less than 200,000 cases by the early 2000s [4]. However, despite these positive features, leprosy persists as a public health concern in some endemic countries, with an estimated 200,000 new cases detected annually worldwide [5].

Leprosy is most prevalent in tropical and subtropical regions, where environmental factors such as temperature and humidity facilitate bacterial transmission. Countries with the highest burden of leprosy include India, Brazil, Indonesia, Madagascar, and several African nations [6]. More than half of all new leprosy cases yearly are from India. Within endemic countries, leprosy tends to cluster in specific geographic areas, often in marginalized and underserved communities with limited access to healthcare services [7].

The epidemiology of leprosy is significantly affected by socioeconomic factors, including poverty, overcrowding, and inadequate sanitation. Individuals living in impoverished conditions are at higher risk of contracting leprosy due to limited access to healthcare, poor nutrition, and substandard living conditions [8]. In addition, social stigma and discrimination associated with leprosy contribute to delayed diagnosis and treatment‐seeking behavior, further perpetuating transmission within affected communities.

Leprosy can affect individuals of all ages and genders, but some age groups may be more susceptible than others. While new cases of leprosy can occur at any age, the disease predominantly affects young adults, with peak incidence observed in the 15‐44 age group [9]. Moreover, there is evidence to suggest a slight male predominance in leprosy cases, although the reasons for this gender disparity are not fully understood.

Risk factors for the transmission and susceptibility of leprosy

Environmental, genetic, and socioeconomic factors influence leprosy transmission. In this context, leprosy is primarily transmitted through prolonged close contact with untreated individuals who have multibacillary forms of the disease [10]. Household contacts, particularly family members, are at higher risk due to prolonged exposure to infectious nasal secretions or skin lesions. Overcrowded and impoverished living environments facilitate the transmission of leprosy. Poor sanitation, inadequate housing, and lack of clean water increase the risk of spreading disease, particularly in densely populated urban slums and rural communities [11].

Some occupations or activities associated with close contact with soil, animals, or vectors may increase the risk of leprosy transmission. Agricultural workers, hunters, and individuals involved in construction or mining activities are at higher risk due to potential exposure to *M. leprae* in the environment [12]. Population movements, migration, and displacement contribute to the spread of leprosy across geographical regions. Migrants from endemic areas may introduce the disease to new communities, while mobility within endemic regions can facilitate the dissemination of infection [13].

Genetic predisposition plays a significant role in determining individual susceptibility to leprosy. Variations in host immune response genes, such as *HLA‐DR *(human leukocyte antigen-DR isotype) and *TNF‐alpha *(tumor necrosis factor-alpha) polymorphisms, influence susceptibility to infection and disease progression [14]. Some ethnic groups and family clusters may exhibit higher rates of leprosy due to genetic susceptibility factors. Impaired cell‐mediated immunity, particularly defects in T‐cell function, predisposes individuals to leprosy infection and progression. Immunosuppressive conditions, such as HIV (human immunodeficiency virus) infection, malnutrition, and immunosuppressive therapy, increase susceptibility to *M. leprae *infection and the development of clinical disease [15]. Leprosy incidence varies by age and gender, with higher rates observed in certain age groups and among males than females. Children and adolescents are at increased risk of developing leprosy, possibly due to immunological immaturity and higher exposure to infectious contacts [16].

Malnutrition and poverty contribute to immune dysfunction and increased susceptibility to infectious diseases, including leprosy. Poor nutritional status, inadequate sanitation, and lack of access to healthcare exacerbate the burden of leprosy in socio‐economically disadvantaged populations. Co‐existing infections, such as tuberculosis and helminthiasis, and underlying comorbidities, such as diabetes mellitus and chronic renal disease, can impair immune function and increase susceptibility to leprosy [17]. Concurrent infections may also exacerbate disease severity and complicate treatment outcomes.

Introduction to the neurological aspect of leprosy and its significance

Leprosy, historically known for its devastating effects on the skin, also can significantly impact the nervous system, leading to a spectrum of neurological manifestations. While the skin lesions of leprosy are often the most visible signs of the disease, neurological complications can result in significant morbidity and disability.

Leprosy primarily affects the peripheral nerves, leading to neurological symptoms and signs. The most common neurological manifestation of leprosy is peripheral neuropathy, characterized by sensory loss, motor weakness, and nerve enlargement. Peripheral neuropathy can exist in the absence of skin lesions. However, it is a rare manifestation of leprosy [18]. Nerve damage in leprosy can occur through direct invasion by *M. leprae*, immune‐mediated mechanisms, or secondary to inflammation and fibrosis [19]. The extent and severity of nerve involvement vary widely among individuals, contributing to the diverse clinical presentations observed in leprosy.

The neurological complications of leprosy have significant clinical implications for affected individuals. Sensory loss and motor impairment can lead to disabilities such as muscle weakness, foot drop, and loss of protective sensation, predisposing individuals to injury and secondary infections. Moreover, autonomic dysfunction in leprosy can manifest as impaired sweating, changes in blood pressure regulation, and gastrointestinal disturbances, further impacting the overall health and well‐being of patients.

Pathophysiology of leprosy

Overview of M. leprae and Its Neurotropism

**Figure 1:**
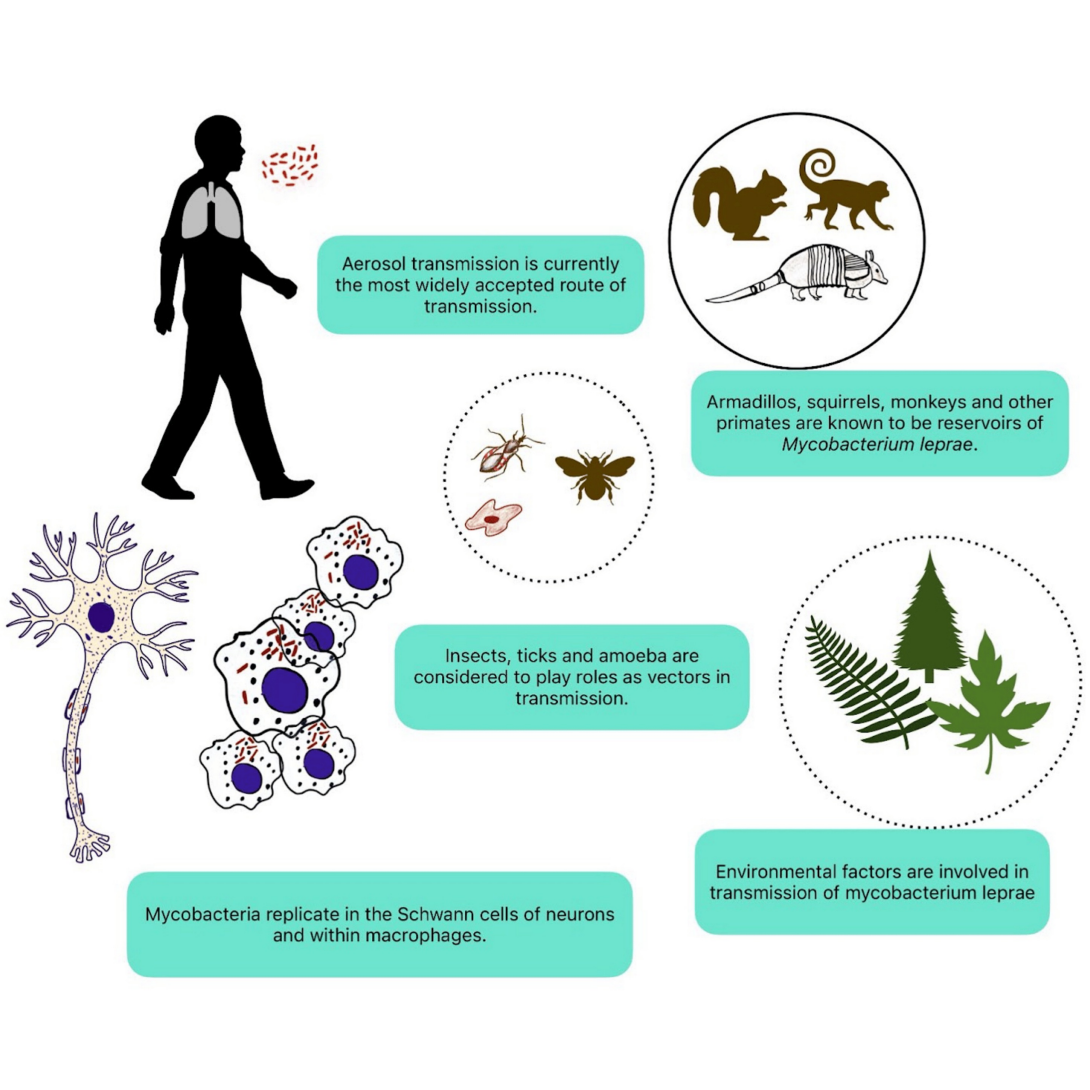
Transmission and neural invasion of Mycobacterium leprae (M. leprae). Aerosol transmission is the most accepted route, although vectors and environmental factors are still being studied. Once they enter the bloodstream, M. leprae replicate in macrophages and reach the central nervous system, where they replicate in Schwann cells. Many animals, such as armadillos and monkeys, are considered reservoirs. Original figure created by Dr. Vishnu Vardhan Byroju.

*M. leprae* possesses unique characteristics that enable it to invade and thrive within the peripheral nerves. Understanding the neurotropism of *M. leprae* is essential for comprehending the pathogenesis of leprosy neuropathy and developing targeted therapeutic interventions (Figure 1) [20].

*M. leprae* is an acid‐fast, obligate intracellular bacterium belonging to the genus *Mycobacterium*. It is one of the smallest free‐living organisms known to infect humans, with a highly streamlined genome and limited metabolic capabilities [21]. Despite its simplicity, *M. leprae* exhibits remarkable resilience, capable of surviving and multiplying within host cells for years to decades. The slow growth rate of *M. leprae* contributes to the chronicity of leprosy infection and the insidious onset of clinical symptoms [22].

*M. leprae* demonstrates a strong preference for neural tissues, particularly the peripheral nerves, where it establishes a preferential niche for replication and persistence. The mechanisms underlying the neurotropism of *M. leprae* are poorly understood. Upon entry into the human body, *M. leprae* infects macrophages and Schwann cells within peripheral nerves, forming characteristic nerve granulomas and destroying neural architecture [23].

*M. leprae *employs various strategies to evade host immune surveillance and establish a chronic infection within the nervous system. These include inhibiting phagolysosome fusion, modulation of host cell signaling pathways, and the production of immunomodulatory molecules [24]. By subverting host immune responses, *M. leprae* evades clearance by the immune system and persists within infected tissues, contributing to the chronicity of leprosy infection and the development of neurological complications.

The neurotropic nature of *M. leprae* plays a central role in the pathogenesis of leprosy neuropathy, leading to the progressive destruction of peripheral nerves and subsequent neurological deficits. Nerve damage in leprosy results from a combination of direct bacterial invasion, immune-mediated inflammation, and neurotoxic effects of bacterial products [25].

Mechanisms of Nerve Damage in Leprosy: Immune Response, Inflammation, and Nerve Injury

Nerve damage in leprosy involves multiple mechanisms involving the interplay between the immune response, inflammation, and direct nerve injury mediated by *M. leprae.* Understanding these mechanisms is crucial for developing effective treatment strategies and mitigating the neurological complications of the disease (Table 1).

**Table 1:** Receptors and molecules related with leprosy neuropathy Original table created by the authors. BDNF: brain-derived neurotrophic factor; Erk1/2: extracellular signal-regulated kinase 1/2; HER: human epidermal growth factor receptor 2; NGF: nerve growth factor; NT-3: neurotrophin-3; NT-4: neurotrophin-4; PGL-I: phenolic glycolipid I; pSLC: progenitor/stem-like cells; TGF-β1: transforming growth factor beta 1; TLR2: Toll-like receptor 2; TNF-α: tumor necrosis factor-alpha.

Receptors and molecules	Mechanism	Reference
Carbon metabolism	*M. leprae* utilizes host glucose pools as the carbon source to biosynthesize the majority of its amino acids.	Borah et al. [26]
Glucose metabolism	*M. leprae* could modulate host cell glucose metabolism to increase the cellular reducing power generation, facilitating glutathione regeneration and, consequently, free radical control.	Medeiros et al. [27]
HER2	*M. leprae* directly binds to and activates *HER2*, bypassing neuregulin–mediated phosphorylation, inducing excessive downstream *Erk1/2* signaling, and subsequently causing demyelination.	Tapinos et al. [28]
Myelin breakdown	Myelin breakdown induces lipid droplet production, providing protective lipid-enriched shelters for *M. leprae* inside Schwann cell.	Mietto et al. [29]
*NT-4, NT-3, NGF* and *BDNF*	*M. leprae* may be involved in neurotrophin regulation that may induce nerve degeneration or repair.	Nogueira et al. [30]
PGL-1	PGL-1 help in *M. leprae* entry and survival in Schwann cell.	Díaz Acosta et al. [31]
	*M. leprae* *PGL-1 *induces macrophage neurotoxic response by inducing reactive oxygen species production, causing axonal and mitochondrial damage that leads to demyelination.	Madigan et al. [32]
pSLC	*pSLC* promotes bacterial spread by direct differentiation to mesenchymal tissues, granuloma-like structures, and subsequent release of bacteria-laden macrophages.	Masaki et al. [33]
	*M. leprae* induces the expression of a variety of genes related to innate immunity in Schwann cell strains in the early stage of infection, even before there were gene modifications associated with reprogramming in *pSLC.*	Masaki et al. [34]
Schwann cells and Schwann cell–axon interactions in co-cultures	Schwan cells maintained fifty percent viability at 33˚C for three weeks and altered morphology and gene expression that encoded cellular adhesion molecules but were capable of cellular interaction.	Hagge et al. [35]
	*M. leprae* propagates a non-myelinating phenotype by inducing demyelination and nerve injury in myelinated Schwann cells in the early phase of infection, a novel bacterial survival strategy in the nervous system.	Rambukkana et al. [36]
*TGF-β1* and *TNF-α*	*M. leprae*-infected Schwann cells undergo phenotypic changes and even death as a result of *TGF-β1*, leading them to secrete extracellular matrix that contributes to progressive nerve fiber loss and fibrosis.	Petito et al. [37]
	Induction of Schwann cell death can provide an effective mechanism of ongoing tissue injury during *M. leprae* infection, which, in turn, may be further modulated by cell-cell interaction and cytokine production.	Oliveira et al. [38]
	*M. leprae *and *TNF* may directly induce Schwann cells to upregulate and secrete *MMPs* regardless of the extent of inflammation in leprous neuropathy.	Oliveira et al. [39]
	ML is capable of contributing to a *TNF*-mediated response by inducing mTNF expression and upregulating *TNFR1*, thus rendering Schwann cells more sensitive to the exogenous *TNF* levels in the nerve.	Andrade et al. [40]
TLR2	*TLR2* ligation induces apoptosis of human Schwann cells and causes nerve damage by the host immune response.	Oliveira et al. [41]

The immune response to *M. leprae* involves both cellular and humoral components. CD4+ T‐helper cells and CD8+ cytotoxic T‐cells recognize *M. leprae* antigens presented by antigen‐presenting cells, leading to the activation of macrophages and the release of pro‐inflammatory cytokines. The immune response to *M. leprae* results in the formation of granulomas within affected tissues, including peripheral nerves. Granulomas are organized structures composed of macrophages, lymphocytes, and other immune cells. While granuloma formation is a host defense mechanism to contain the infection, it can also contribute to tissue damage and nerve dysfunction [42].

The release of pro‐inflammatory cytokines such as *tumor necrosis factor‐α, interleukin‐1,* and *interleukin‐6* contributes to tissue inflammation and damage in leprosy [43]. These cytokines stimulate immune cell activation and recruitment, leading to the perpetuation of inflammation within affected nerves. Inflammatory processes in leprosy generate reactive oxygen species and reactive nitrogen species, leading to oxidative stress within nerve tissues. Oxidative stress contributes to nerve damage through lipid peroxidation, protein oxidation, and DNA damage, further exacerbating neuronal injury [44].

*M. leprae* has a tropism for neural tissues, allowing it to invade and replicate within Schwann cells, macrophages, and endoneurial cells within peripheral nerves. Direct bacterial invasion leads to the destruction of nerve architecture, disruption of myelin sheaths, and axonal degeneration. *M. leprae* produces various neurotoxic products, including glycolipids, lipoproteins, and cell wall components, which can damage nerve cells and impair neuronal function. These neurotoxic products induce inflammation, oxidative stress, and apoptosis within affected nerves, contributing to nerve damage and dysfunction [45].

## Review

Clinical spectrum of manifestations

Spectrum of Clinical Presentations: Tuberculoid, Lepromatous, and Borderline Forms

**Figure 2:**
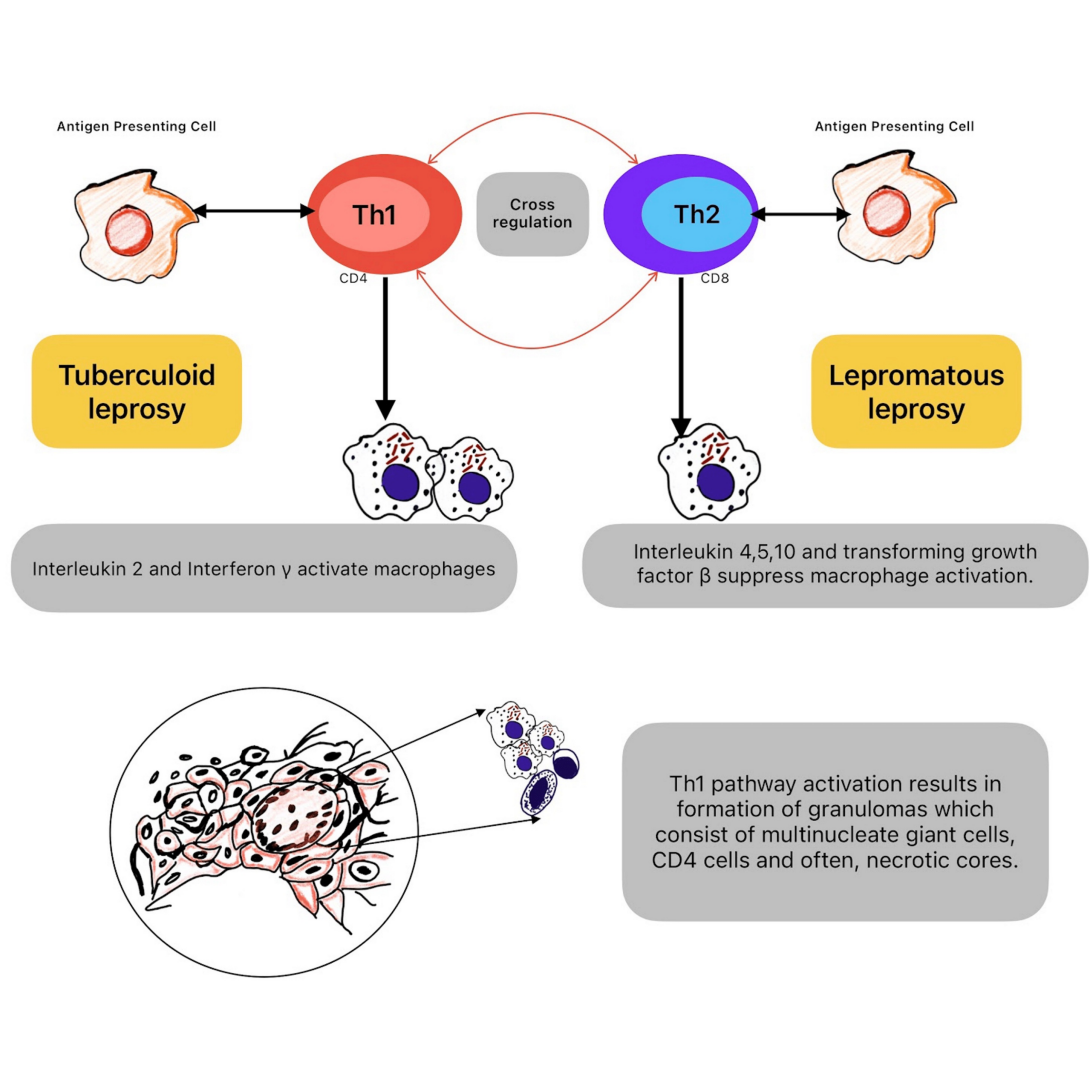
Th1 and Th2 responses in leprosy. Antigen-presenting cells activate Th1 cells, resulting in macrophage activation and granuloma formation. This triggers a tuberculoid form of leprosy, which is less severe. When the Th2 pathway is activated, macrophage activation is limited, and disseminated disease develops, termed lepromatous leprosy. Original figure created by Dr. Vishnu Vardhan Byroju.

The spectrum of leprosy clinical presentations encompasses various manifestations, classified into three primary forms: tuberculoid, lepromatous, and borderline. These classifications reflect the spectrum of immune response to *M. leprae* infection and the severity of disease manifestations (Figure 2) [46].

Tuberculoid leprosy represents the milder end of the spectrum. It is characterized by a localized and robust cell‐mediated immune response against *M. leprae*, in which hypopigmented or erythematous macules or plaques have well‐defined borders. Usually, it affects only a few peripheral nerves, leading to focal sensory loss and nerve thickening. Loss of sensation within the affected skin patches. Alopecia within the lesions due to nerve damage. Palpable peripheral nerve enlargement due to granuloma formation and inflammation. Low or absent bacterial load in skin lesions, with few or no *acid-fast bacilli (AFB) *observed on skin smears. Lepromatous leprosy represents the more severe end of the spectrum. It is characterized by an ineffective immune response, allowing uncontrolled bacterial proliferation. It led to widespread and symmetrical involvement with diffuse infiltration and nodular lesions and thickened, infiltrated skin over the face and ears, giving a leonine appearance. Extensive nerve involvement causes sensory, motor, and autonomic deficits. Profound and generalized sensory loss, including pain, touch, and temperature sensation deficits. Muscle weakness and wasting due to nerve damage, leading to clawing of hands and foot drop. High bacterial load in skin lesions, with numerous *AFB* observed on skin smears. Borderline leprosy represents an intermediate form between tuberculoid and lepromatous leprosy, exhibiting features of both polar forms. Variable and patchy skin involvement, with a mixture of hypo‐ and erythematous lesions. Variable nerve involvement, with features of both tuberculoid and lepromatous neuropathy. Sensory loss and motor weakness may vary in severity and distribution. Moderate bacterial load in skin lesions, with variable numbers of *AFB*, observed on skin smears. Moreover, borderline leprosy is further classified into borderline tuberculoid (BT), borderline borderline (BB), and borderline lepromatous (BL) based on the predominance of features from either end of the spectrum [47].

Peripheral Nerve Involvement: Sensory and Motor Deficits and Nerve Enlargement

Leprosy is renowned for its preference to affect the peripheral nerves, leading to sensory and motor deficits. One of the hallmark features of leprosy neuropathy is nerve enlargement, known as neuritis. The prevalence of neuropathic pain among leprosy patients is still poorly reported and probably underestimated (Table 2).

**Table 2:** Studies on the prevalence of leprosy neuropathic pain Original table created by the authors.

Reference	Country, year (prevalence)	Comments
Lasry-Levy et al. [48]	India, 2011 (21.78%)	Neuropathic pain and nerve enlargement persistence association were observed.
Haroun et al. [49]	Ethiopia, 2012 (60%)	Neuritis-related pain may occur earlier during the course of the disease.
Raicher et al. [50]	Brazil, 2016 (78.88%)	A significant percentage of the individuals (83%) of patients had already completed antibiotic treatment.

Leprosy commonly presents with sensory deficits, ranging from mild hypoesthesia to complete anesthesia. Sensory impairment typically affects the distal extremities first, progressing proximally as the disease advances. Loss of sensation predisposes individuals to injuries, burns, and secondary infections, particularly in areas prone to trauma. Some individuals with leprosy may experience abnormal sensations such as tingling, burning, or numbness, known as paresthesia. Paresthesia often accompanies sensory loss and may be distressing for affected individuals. Leprosy neuropathy can lead to motor deficits, including muscle weakness and atrophy, particularly in the hands and feet. Weakness may result from denervation of muscles due to nerve damage, leading to impaired motor function and coordination. Muscle weakness contributes to functional disability and may lead to deformities if left untreated. In advanced cases of leprosy neuropathy, weakness of the muscles that dorsiflex the foot can result in foot drop. Foot drops can alter gait structure and increase the risk of falls and injuries [51].

Neuritis is a characteristic feature of leprosy neuropathy observed in affected nerves. They may become tender to palpation and exhibit palpable thickening due to inflammation and edema. Neuritis is often asymmetric and may involve multiple peripheral nerves simultaneously. Neuritis in leprosy tends to affect nerves in cooler areas of the body, such as the elbows, knees, wrists, and ankles. The cooler temperature may facilitate bacterial growth and exacerbate inflammatory responses within affected nerves [52].

Leprosy Neuropathy: Classification and Clinical Features

Classification systems for leprosy neuropathy help clinicians to guide treatment decisions. In this context, Ridley and Jopling proposed a widely used classification system based on clinical, histological, and immunological criteria [53]. Classifies leprosy into five main subtypes: indeterminate, tuberculoid, borderline tuberculoid, borderline, and lepromatous. Each subtype represents a distinct spectrum of disease severity and immune response to* M. leprae*, with implications for prognosis and treatment. The Madrid classification system categorizes leprosy neuropathy based on nerve function impairment and nerve damage. It also classifies leprosy neuropathy into three main categories, namely, neuritis, neuropathy without visible deformity, and neuropathy with visible deformity, and provides a practical framework for assessing nerve function and guiding treatment decisions in leprosy neuropathy.

Diagnostic approaches

Clinical Assessment and Diagnosis

Early recognition of neurological involvement in leprosy is crucial for preventing irreversible nerve damage and mitigating long‐term disability. A comprehensive clinical assessment focusing on signs and symptoms suggestive of neurological deficits is essential for timely diagnosis and appropriate management. Nerve conduction studies (NCS) may demonstrate abnormalities in nerve conduction velocity, compound muscle action potential, and sensory nerve action potential, indicative of nerve dysfunction. Nerve biopsy, skin smears, and imaging techniques play crucial roles in diagnosing leprosy neuropathy, offering valuable insights into the pathology and localization of the disease. A nerve biopsy is a useful diagnostic tool for assessing nerve pathology and confirming the presence of *M. leprae* within affected nerves [54]. During nerve biopsy, a small portion of an affected nerve, typically a superficial sensory nerve, is surgically excised under local anesthesia. Histological analysis of nerve biopsy specimens allows for detecting characteristic histopathological features of leprosy, including granulomas, *AFB*, and nerve damage. Nerve biopsy findings help classify leprosy neuropathy according to the Ridley‐Jopling classification system, guiding treatment decisions and prognostic assessments [55].

Slit skin smear examination is a rapid and minimally invasive diagnostic test used to detect *AFB* in skin lesions and peripheral nerves. A small incision is made in a skin lesion or affected nerve, and the exudate is collected on a glass slide. To visualize *AFB*, the sample is stained using the Ziehl‐Neelsen or Fite‐Faraco staining method [56]. Positive skin smears confirm the presence of *M. leprae* infection and aid in the classification of leprosy based on the bacterial index, a semi‐quantitative measure of bacterial load. Serial skin smear examinations are performed during treatment to monitor the efficacy of antimicrobial therapy and assess bacterial clearance. Ultrasonography (USG) is a non‐invasive imaging modality used to evaluate nerve morphology, detect nerve enlargement, and assess nerve damage in leprosy neuropathy [57]. USG allows for real‐time visualization of nerve structures and may aid in diagnosing and monitoring leprosy neuropathy. Chen et al. observed that USG features of leprosy disease contained segmental and asymmetrical patterns of increased cross-sectional area (CSA). The electrophysiological findings introduced no response, decreased conduction velocities, delayed latency, and reduced amplitude in influenced nerves. Nerve pathological features implied *AFB* staining, axon and myelin sheath devastation, hyperplasia of Schwann cells along with the absence of macrophages, fibrosis, and edema [58]. Magnetic resonance imaging (MRI) provides detailed anatomical images of peripheral nerves and surrounding tissues, allowing for the assessment of nerve enlargement, inflammation, and damage in leprosy neuropathy. MRI is beneficial for evaluating deep‐seated nerves and assessing the extent of nerve involvement in leprosy. NCS measures the electrical conduction of peripheral nerves and helps evaluate nerve function in leprosy neuropathy. NCS can detect abnormalities in nerve conduction velocity and amplitude, providing valuable information about the extent and severity of nerve damage.

Challenges in Early Detection and Differential Diagnosis

Diverse clinical presentations, overlapping symptoms with other neurological conditions, and the persistence of social stigma surrounding the disease are some of the challenges for the diagnosis of leprosy. Addressing these challenges is crucial for initiating timely treatment and preventing long‐term disability.

Leprosy neuropathy can present with a wide range of clinical manifestations, including sensory deficits, motor weakness, autonomic dysfunction, and skin lesions. However, these symptoms may be subtle or nonspecific, leading to delays in diagnosis. Atypical presentations, such as pure neural leprosy without cutaneous involvement or paucibacillary leprosy with few or no visible skin lesions, further complicate early detection and may be mistaken for other neurological or dermatological conditions [59]. Limited awareness among healthcare providers and the general population about the diverse manifestations of leprosy neuropathy contributes to diagnostic delays and underreporting of cases [60]. In regions where leprosy is considered rare or eliminated, healthcare professionals may lack familiarity with the clinical features of leprosy neuropathy, leading to misdiagnosis or delayed referral to specialized services. The enduring social stigma associated with leprosy discourages affected individuals from seeking medical attention and disclosing their symptoms, further delaying diagnosis and treatment. Fear of discrimination within communities perpetuates the concealment of symptoms and hinders early detection efforts. The symptoms of leprosy neuropathy may overlap with other neurological conditions, including peripheral neuropathies, spinal cord disorders, and autoimmune diseases. Differential diagnosis requires careful consideration of clinical features, nerve involvement patterns, and ancillary tests to distinguish leprosy neuropathy from other neurologic disorders.

Access to diagnostic resources, such as nerve biopsy, skin smears, and specialized imaging techniques, may be limited in resource‐constrained settings where leprosy is endemic. Lack of trained personnel, equipment, and infrastructure for performing and interpreting diagnostic tests contributes to diagnostic delays and compromises the accuracy of diagnosis [61]. Subclinical or asymptomatic infections with *M. leprae* may go unrecognized, particularly in individuals with low bacterial loads and preserved nerve function. Screening high‐risk populations, such as household contacts of leprosy patients and individuals living in endemic regions, is essential for detecting subclinical infections and preventing transmission [62].

Management strategies

Multidrug Therapy and Its Efficacy in Preventing Neurological Sequelae

Multidrug therapy (MDT) is the cornerstone of leprosy treatment, aiming not only to cure the infection but also to prevent the progression of neurological sequelae and disability [63]. MDT combines multiple antibiotics to target *M. leprae* effectively and reduce the risk of treatment failure and relapse. MDT consists of a combination of antibiotics, usually rifampicin, dapsone, and clofazimine, which target different stages of the leprosy infection. Rifampicin is bactericidal and kills actively dividing *M. leprae*, while dapsone and clofazimine are bacteriostatic and prevent bacterial replication [64]. The synergistic action of these antibiotics in MDT effectively eliminates *M. leprae* from the body, reducing the bacterial load and preventing further nerve damage [65]. Early diagnosis and initiation of MDT are crucial for preventing neurological sequelae in leprosy [66]. Prompt treatment with MDT helps arrest the progression of the disease, preventing irreversible nerve damage and disability. The WHO recommends initiating MDT as soon as leprosy is diagnosed, regardless of the clinical subtype or severity of symptoms. MDT regimens are designed to provide a prolonged course of treatment, typically spanning six to 12 months for paucibacillary leprosy and 12 to 24 months for multibacillary leprosy [67]. Completing the entire course of MDT reduces the risk of relapse and recurrence of leprosy, which could lead to further nerve damage and neurological sequelae [68]. In addition to its antibacterial effects, MDT has anti‐inflammatory properties that help reduce inflammation and prevent nerve damage in leprosy. MDT mitigates nerve inflammation and preserves nerve function by suppressing the inflammatory response against M. leprae. Steroids are frequently prescribed for leprosy neuropathy, but there are no studies supporting this treatment choice (Table 3). Some limitations are that the studies were retrospective, observational, and did not compare the same group of leprosy patients.

**Table 3:** Studies using steroids in leprosy neuropathy Original table created by the authors.

Reference	Country, year	Population enrolled	Intervention
Van Brakel et al. [69]	Nepal, 2003	75	16-week standard prednisolone regime
Richardus et al. [70]	Nepal, 2003	92	16-week standard prednisolone regime
Marlowe et al. [71]	Nepal, 2004	40	12 weeks of azathioprine and eight weeks of prednisolone compared with 12 weeks of prednisolone alone
Rao et al. [72]	India, 2006	334	Three prednisolone regimes: 3.5 g over 5 months, 2.31 g over five months, 2.94 g over three months
Garbino et al. [73]	Brazil, 2008	21	Prednisone 120 mg daily initially compared with 60 mg daily initially for controls; tapered variably
Walker et al. [74]	Nepal, 2011	42	Methylprednisolone 1g intravenously for three days followed by prednisolone 40 mg daily reducing over 109 days to 0 versus prednisolone 40 mg daily reducing over 112 days

Rehabilitation Interventions for Neuropathic Pain and Disabilities

Rehabilitation interventions play a crucial role in managing neuropathic pain and disabilities associated with leprosy neuropathy, aiming to improve functional independence, quality of life, and overall well‐being for affected individuals. Physiotherapy focuses on maintaining or improving joint mobility through passive and active range of motion exercises. This helps prevent contractures and stiffness commonly observed in individuals with leprosy neuropathy [75]. Specific muscle groups weakened by leprosy neuropathy are targeted with strengthening exercises to improve muscle strength and functional capacity. Individuals with foot drop or other gait abnormalities receive gait training to improve walking mechanics, balance, and stability. Assistive devices such as orthoses or braces may be prescribed to facilitate walking. Balance and coordination exercises help individuals with sensory and motor deficits maintain stability and reduce the risk of falls [76].

Rehabilitation professionals provide education and counseling to individuals with neuropathic pain, helping them understand the nature of their pain and develop coping strategies. Modalities such as transcutaneous electrical nerve stimulation, heat therapy, cold therapy, and massage may alleviate neuropathic pain and improve comfort [77]. Rehabilitation specialists collaborate with healthcare providers to optimize the pharmacological management of neuropathic pain, including the use of analgesics, antidepressants, anticonvulsants, and topical agents. In addition, individuals with altered sensation receive sensory re‐education to desensitize hypersensitive areas and improve tactile discrimination. Sensory re‐education programs include activities to improve texture discrimination and enhance sensory feedback, facilitating the performance of fine motor tasks. Moreover, rehabilitation professionals offer counseling and facilitate peer support groups to address psychosocial issues associated with leprosy neuropathy, such as stigma, isolation, depression, and anxiety. Individuals with disabilities receive vocational rehabilitation services to explore employment opportunities, develop job skills, and achieve economic independence [78].

Surgical Interventions for Nerve Damage and Deformities

Surgical procedures address the consequences of nerve damage, muscle weakness, joint contractures, and sensory loss associated with leprosy neuropathy. Nerve compression due to thickened fascial bands or scar tissue can lead to neuropathic pain, sensory deficits, and motor dysfunction. Surgical decompression involves releasing the constricting structures around affected nerves to relieve pressure and improve nerve function. This may include neurolysis, neurolysis with nerve grafting, or neurolysis with tendon transfer. Muscle weakness and paralysis resulting from nerve damage in leprosy neuropathy can lead to deformities such as claw, hand, or foot drop. A tendon transfer involves transferring a healthy tendon from an unaffected muscle to a paralyzed or weakened muscle to restore function and improve joint alignment. This helps correct deformities and improve hand and foot function [79].

Joint deformities, such as contractures and malalignments, may develop due to muscle imbalances and sensory loss in leprosy neuropathy. Corrective osteotomy involves surgically cutting and realigning the bones to correct joint deformities and improve joint function and stability. This may be performed in the hands, feet, or other affected joints to restore the range of motion and prevent further deterioration. Soft tissue contractures, such as plantar fascial or flexion contractures in the fingers, can lead to gait abnormalities and impaired hand function. Soft tissue release involves surgically releasing tight or shortened soft tissues, such as tendons, ligaments, or fascia, to improve joint mobility and reduce contractures. This may be combined with tendon lengthening or transfer to optimize functional outcomes [80].

Chronic ulcerations and tissue loss resulting from sensory loss and autonomic dysfunction in leprosy neuropathy may require surgical intervention to promote wound healing and prevent infection. Skin grafting and flap surgery involve transplanting healthy skin from donor sites to cover ulcerated or damaged areas, promoting tissue regeneration and wound closure. This helps prevent complications such as infection and facilitates rehabilitation. Severe nerve damage and loss of sensation may necessitate peripheral nerve surgery to restore sensory function and prevent further deterioration. Peripheral nerve surgery includes nerve repair, nerve grafting, or nerve transfer to repair damaged nerves, restore sensation, and improve nerve conduction. This may be performed in conjunction with other surgical interventions to optimize outcomes [81].

Global burden

As of recent data available, the global burden of leprosy remains significant, although the disease has been largely controlled in many regions. According to the WHO and the Global Leprosy Programme, approximately 200,000 new cases of leprosy were reported worldwide in 2020. This number has remained relatively stable over the past few years. Leprosy continues to be endemic in several countries, with the highest burden reported in India, Brazil, and Indonesia. These countries account for the majority of new cases globally. In addition, leprosy disproportionately affects marginalized and vulnerable populations, including those living in poverty, rural areas, and areas with limited access to healthcare [82]. Despite significant progress in the diagnosis and treatment of leprosy, challenges such as delayed detection, social stigma, and discrimination persist, hindering efforts to eliminate the disease. Early detection and prompt treatment remain essential for preventing disabilities and reducing transmission [83].

Efforts to control leprosy are ongoing, with initiatives focusing on improving surveillance, strengthening healthcare systems, enhancing access to MDT, and promoting social inclusion and rehabilitation for affected individuals [84]. Through sustained efforts and collaboration among governments, healthcare organizations, and international partners, the global burden of leprosy continues to decline. Still, sustained efforts are necessary to achieve the goal of eliminating leprosy as a public health problem. While specific data on the prevalence of leprosy neuropathy globally may be limited, it is well‐established that neurological complications are common among individuals affected by leprosy, particularly in regions where the disease is endemic. The prevalence of leprosy neuropathy is directly related to the overall burden of leprosy in a given population [85]. The clinical spectrum of leprosy ranges from paucibacillary to multibacillary. Multibacillary leprosy is associated with a higher risk of neurological complications due to the higher bacterial load and more significant immune response.

Psychological and social implications of neurological deficits in leprosy

The stigma associated with leprosy, historically rooted in misconceptions and fear, often extends to individuals with visible signs of neurological deficits, such as deformities and disabilities. Negative attitudes and discrimination toward individuals with leprosy neuropathy may lead to social rejection, exclusion from community activities, and difficulty accessing employment and education opportunities [86].

Neurological deficits, such as sensory loss, muscle weakness, and deformities, can impact an individual’s self‐esteem and confidence, mainly if they affect physical appearance or functional abilities. Feelings of inadequacy, shame, and embarrassment may arise from the visible signs of leprosy neuropathy, leading to diminished self‐worth and reluctance to engage in social interactions [87].

Coping with the challenges of living with neurological deficits in leprosy can contribute to emotional distress, anxiety, depression, and other mental health issues. Fear of social rejection, anticipation of future disability, and uncertainty about the progression of the disease may exacerbate psychological distress among affected individuals and their families. The visible signs of leprosy neuropathy, such as deformities and disabilities, may lead to social isolation and loneliness as individuals experience rejection and avoidance by others. Withdrawal from social activities, loss of social support networks, and limited opportunities for meaningful social interactions can further exacerbate feelings of isolation and loneliness [88].

Neurological deficits in leprosy can strain relationships with family members and friends, particularly if they require caregiving or impose financial burdens on the household. Family members may experience emotional distress and feelings of guilt or responsibility for the affected individual’s condition, leading to conflicts and disruptions in family dynamics. Individuals with leprosy neuropathy may encounter barriers to accessing healthcare services, including stigma‐related discrimination, lack of financial resources, and limited availability of specialized care [89]. Barriers to rehabilitation services, such as physiotherapy, occupational therapy, and psychological support, can further exacerbate the impact of neurological deficits on psychological well‐being and social integration.

Future directions and research opportunities

Emerging Trends in Neuroimaging and Molecular Diagnostics for Leprosy Neuropathy

Advanced MRI techniques, such as diffusion tensor imaging and magnetic resonance neurography, enable high‐resolution visualization of peripheral nerves and provide insights into nerve morphology, integrity, and pathology in leprosy neuropathy. PET imaging with radiolabeled tracers can provide valuable information about neuroinflammatory processes, metabolic activity, and neurodegeneration in leprosy neuropathy, facilitating early detection and monitoring of disease progression [90].

Transcriptomic analysis of peripheral nerve tissue or blood samples can identify gene expression patterns associated with leprosy neuropathy, providing insights into disease pathogenesis, immune response, and potential biomarkers for diagnosis and prognosis. Characterization of the skin and gut microbiome in individuals with leprosy neuropathy may uncover microbial signatures associated with disease susceptibility, progression, and treatment response, offering novel targets for intervention and personalized therapy. Proteomic and metabolomic approaches enable the identification of biomarkers, such as cytokines, chemokines, and metabolic intermediates, that reflect disease activity, nerve damage, and treatment response in leprosy neuropathy, facilitating early diagnosis and targeted therapy [91].

AI‐driven algorithms and machine learning techniques are applied to neuroimaging data, molecular profiles, and clinical data to develop predictive models, diagnostic classifiers, and treatment algorithms for leprosy neuropathy. AI‐based image analysis tools can automate the quantification of nerve morphology, lesion burden, and disease progression in neuroimaging studies, enhancing diagnostic accuracy and efficiency [92].

Rapid diagnostic tests based on molecular and immunological markers are being developed for point‐of‐care detection of leprosy neuropathy in resource‐limited settings, enabling early intervention and reducing diagnostic delays. Portable neuroimaging devices and smartphone‐based applications for image acquisition and analysis are under development, facilitating remote screening, monitoring, and triage of individuals with suspected neurological involvement in leprosy [93]. Integrating multi‐omics data, including genomics, transcriptomics, proteomics, metabolomics, and microbiomics, offers a comprehensive understanding of the molecular mechanisms underlying leprosy neuropathy and identifies potential therapeutic targets and biomarkers for precision medicine approaches.

Potential Targets for Novel Therapeutic Interventions

Modulating macrophage activation towards an anti‐inflammatory *M2* phenotype may reduce tissue damage and inflammation in leprosy lesions. Targeting *regulatory T‐cells* or *T‐helper cell* subsets implicated in leprosy pathogenesis may help restore immune balance and prevent disease progression. Blockade of pro‐inflammatory cytokines or cytokine receptors may dampen immune‐mediated tissue damage and neuropathy [94].

Developing new antibiotics with enhanced efficacy against *M. leprae*, including drugs targeting dormant or persistent bacilli, may improve treatment outcomes and shorten therapy duration. Identifying compounds with potent bactericidal activity against *M. leprae*, particularly in the intracellular environment, is crucial for eliminating bacterial reservoirs and preventing relapse. Adoptive cell transfer of autologous or engineered immune cells, such as *dendritic* or *cytotoxic T cells*, may enhance host defense mechanisms against *M. leprae* and promote bacterial clearance. Administration of monoclonal antibodies or immunomodulatory proteins targeting specific immune checkpoints or inflammatory pathways may modulate immune responses and mitigate tissue damage in leprosy [95].

Administration of neurotrophic factors or small molecules that promote nerve regeneration and repair may attenuate nerve damage and prevent neurological sequelae in leprosy neuropathy. Anti‐inflammatory agents targeting neuroinflammatory pathways, such as glial activation or cytokine‐mediated neurotoxicity, may preserve nerve function and prevent disability [96].

Co‐administration of adjunctive therapies, such as vitamins, antioxidants, or probiotics, may augment host immune responses, enhance treatment tolerance, and reduce side effects. Stratification of patients based on biomarker profiles, genetic polymorphisms, or disease severity may facilitate personalized treatment strategies tailored to individual needs and treatment responses [97].

Multi‐drug therapy has reduced the burden of leprosy, but elimination still requires better use of existing tools and the development of effective vaccines. Dasgupta et al. review evaluated the clinical efficacy, immunogenicity, and safety of leprosy vaccines through 12 selected studies out of 2,163 retrieved from various databases. Eight studies focused on prophylactic vaccination, while four examined therapeutic use. The therapeutic leprosy vaccine showed significant protection with Ramu’s score of ‐3.06 (95% CI: ‐3.96 to ‐2.16), while the bacterial index was insignificant (‐0.26, 95% CI: ‐1.54 to 1.03). In the prophylactic analysis, a pooled relative risk of 0.61 (95% CI: 0.41-0.91) indicated significant effectiveness compared to placebo. *Mw/Mycobacterium welchii/MIP*, *BCG* vaccine, and rifampicin were protective in recipients [98].

## Conclusions

This manuscript has provided a comprehensive exploration of the neurological manifestations of leprosy, offering valuable clinical insights and implications for diagnosis, management, and understanding of the disease. The epidemiological overview underscores the continued global burden of leprosy and highlights the importance of early detection and intervention to prevent neurological sequelae. We have examined the mechanisms of nerve damage in leprosy, including immune response, inflammation, and nerve injury, shedding light on the complex interplay between the host immune system and *M. leprae.* Peripheral nerve involvement, characterized by sensory and motor deficits, nerve enlargement, and deformities, has been extensively studied. Moreover, the role of neuroimaging, nerve biopsy, and molecular diagnostics in enhancing diagnostic accuracy and guiding therapeutic decisions was discussed. The manuscript has also addressed the psychological and social implications of neurological deficits in leprosy, emphasizing the importance of holistic care and support to mitigate stigma, promote social inclusion, and improve the quality of life for affected individuals. Furthermore, emerging neuroimaging and molecular diagnostics trends offer promising avenues for advancing our understanding of leprosy neuropathy and developing novel therapeutic interventions.

## References

[1] Santacroce L, Del Prete R, Charitos IA, Bottalico L (2021). Mycobacterium leprae: a historical study on the origins of leprosy and its social stigma. Infez Med.

[2] Montes N, Jáuregui C, Dinarès R (2024). Tracing leprosy: the paleopathological study of the individuals excavated from the Sant Llàtzer leprosarium in Barcelona, Spain (12th-18th c.). Int J Paleopathol.

[3] Ghosh S, Chaudhuri S (2015). Chronicles of Gerhard-Henrik Armauer Hansen's life and work. Indian J Dermatol.

[4] Dua A (2005). Programmes for the control of leprosy, tuberculosis and malaria. Burden of Disease in India.

[5] Blok DJ, De Vlas SJ, Richardus JH (2015). Global elimination of leprosy by 2020: are we on track?. Parasit Vectors.

[6] (2021). Towards zero leprosy. Global leprosy (‎Hansen’s Disease)‎ strategy 2021-2030. https://www.who.int/publications/i/item/9789290228509.

[7] Weng X, Xing Y, Liu J (2013). Molecular, ethno-spatial epidemiology of leprosy in China: novel insights for tracing leprosy in endemic and non endemic provinces. Infect Genet Evol.

[8] Bhadra S (2022). Leprosy a life changing experience to live ostracized: psychological issues and well-being. Handbook of Health and Well-Being: Challenges, Strategies and Future Trends.

[9] Odriozola EP, Quintana AM, González V, Pasetto RA, Utgés ME, Bruzzone OA, Arnaiz MR (2017). Towards leprosy elimination by 2020: forecasts of epidemiological indicators of leprosy in Corrientes, a province of northeastern Argentina that is a pioneer in leprosy elimination. Mem Inst Oswaldo Cruz.

[10] Internationalis H (2009). Current literature in leprosy. Hansen Int.

[11] Emerson LE, Anantharam P, Yehuala FM, Bilcha KD, Tesfaye AB, Fairley JK (2020). Poor WASH (water, sanitation, and hygiene) conditions are associated with leprosy in North Gondar, Ethiopia. Int J Environ Res Public Health.

[12] Stone AC, Wilbur AK, Buikstra JE, Roberts CA (2009). Tuberculosis and leprosy in perspective. Am J Phys Anthropol.

[13] Murto C, Chammartin F, Schwarz K, da Costa LM, Kaplan C, Heukelbach J (2013). Patterns of migration and risks associated with leprosy among migrants in Maranhão, Brazil. PLoS Negl Trop Dis.

[14] Jarduli LR, Sell AM, Reis PG (2013). Role of HLA, KIR, MICA, and cytokines genes in leprosy. Biomed Res Int.

[15] Barroso DH, Brandão JG, Andrade ES (2021). Leprosy detection rate in patients under immunosuppression for the treatment of dermatological, rheumatological, and gastroenterological diseases: a systematic review of the literature and meta-analysis. BMC Infect Dis.

[16] Santos SD, Penna GO, Costa Mda C, Natividade MS, Teixeira MG (2016). Leprosy in children and adolescents under 15 years old in an urban centre in Brazil. Mem Inst Oswaldo Cruz.

[17] Rakotoarisaona MF, Razafimaharo TI, Sendrasoa FA (2024). Coinfection with leprosy and tuberculosis: a case series in Malagasy patients. Infect Drug Resist.

[18] Spitz CN, Pitta IJ, Andrade L (2024). Case report: myelitis and ganglionitis, an atypical presentation of Hansen's disease. Front Med (Lausanne).

[19] Cabral N, de Figueiredo V, Gandini M (2022). Modulation of the response to Mycobacterium leprae and pathogenesis of leprosy. Front Microbiol.

[20] Shetty V, Antia N, Jacobs J (1988). The pathology of early leprous neuropathy. J Neurol Sci.

[21] Calderone A, Aloisi MC, Casella C, Fiannacca S, Cosenza B, Quartarone A, Calabrò RS (2024). The neurological impact of leprosy: manifestations and treatment approaches. Neurol Int.

[22] Bhat RM, Prakash C (2012). Leprosy: an overview of pathophysiology. Interdiscip Perspect Infect Dis.

[23] Spierings E, De Boer T, Zulianello L, Ottenhoff TH (2000). Novel mechanisms in the immunopathogenesis of leprosy nerve damage: the role of Schwann cells, T cells and Mycobacterium leprae. Immunol Cell Biol.

[24] Silva BJ, Barbosa MG, Andrade PR (2017). Autophagy is an innate mechanism associated with leprosy polarization. PLoS Pathog.

[25] Aarão TL, de Sousa JR, Falcão AS, Falcão LF, Quaresma JA (2018). Nerve growth factor and pathogenesis of leprosy: review and update. Front Immunol.

[26] Borah K, Girardi KD, Mendum TA (2019). Intracellular Mycobacterium leprae utilizes host glucose as a carbon source in Schwann cells. mBio.

[27] Medeiros RC, Girardi KD, Cardoso FK (2016). Subversion of Schwann cell glucose metabolism by Mycobacterium leprae. J Biol Chem.

[28] Tapinos N, Ohnishi M, Rambukkana A (2006). ErbB2 receptor tyrosine kinase signaling mediates early demyelination induced by leprosy bacilli. Nat Med.

[29] Mietto BS, de Souza BJ, Rosa PS, Pessolani MC, Lara FA, Sarno EN (2020). Myelin breakdown favours Mycobacterium leprae survival in Schwann cells. Cell Microbiol.

[30] Nogueira MR, Amôr NG, Michellin LB (2020). Effect of Mycobacterium leprae on neurotrophins expression in human Schwann cells and mouse sciatic nerves. Mem Inst Oswaldo Cruz.

[31] Díaz Acosta CC, Dias AA, Rosa TL (2018). PGL I expression in live bacteria allows activation of a CD206/PPARγ cross-talk that may contribute to successful Mycobacterium leprae colonization of peripheral nerves. PLoS Pathog.

[32] Madigan CA, Cambier CJ, Kelly-Scumpia KM (2017). A macrophage response to Mycobacterium leprae phenolic glycolipid initiates nerve damage in leprosy. Cell.

[33] Masaki T, Qu J, Cholewa-Waclaw J, Burr K, Raaum R, Rambukkana A (2013). Reprogramming adult Schwann cells to stem cell-like cells by leprosy bacilli promotes dissemination of infection. Cell.

[34] Masaki T, McGlinchey A, Cholewa-Waclaw J, Qu J, Tomlinson SR, Rambukkana A (2014). Innate immune response precedes Mycobacterium leprae-induced reprogramming of adult Schwann cells. Cell Reprogram.

[35] Hagge DA, Oby Robinson S, Scollard D, McCormick G, Williams DL (2002). A new model for studying the effects of Mycobacterium leprae on Schwann cell and neuron interactions. J Infect Dis.

[36] Rambukkana A, Zanazzi G, Tapinos N, Salzer JL (2002). Contact-dependent demyelination by Mycobacterium leprae in the absence of immune cells. Science.

[37] Petito RB, Amadeu TP, Pascarelli BM, Jardim MR, Vital RT, Antunes SL, Sarno EN (2013). Transforming growth factor-β1 may be a key mediator of the fibrogenic properties of neural cells in leprosy. J Neuropathol Exp Neurol.

[38] Oliveira RB, Sampaio EP, Aarestrup F (2005). Cytokines and Mycobacterium leprae induce apoptosis in human Schwann cells. J Neuropathol Exp Neurol.

[39] Oliveira AL, Antunes SL, Teles RM (2010). Schwann cells producing matrix metalloproteinases under Mycobacterium leprae stimulation may play a role in the outcome of leprous neuropathy. J Neuropathol Exp Neurol.

[40] Andrade PR, Jardim MR, da Silva AC (2016). Inflammatory cytokines are involved in focal demyelination in leprosy neuritis. J Neuropathol Exp Neurol.

[41] Oliveira RB, Ochoa MT, Sieling PA, Rea TH, Rambukkana A, Sarno EN, Modlin RL (2003). Expression of Toll-like receptor 2 on human Schwann cells: a mechanism of nerve damage in leprosy. Infect Immun.

[42] Narayanan R (1988). Immunopathology of leprosy granulomas--current status: a review. Lepr Rev.

[43] Nery JA, Bernardes Filho F, Quintanilha J, Machado AM, Oliveira Sde S, Sales AM (2013). Understanding the type 1 reactional state for early diagnosis and treatment: a way to avoid disability in leprosy. An Bras Dermatol.

[44] Potdar A, Dantuma D, Preuss C, Pathak Y (2018). Pharmacology and pharmacokinetics of natural antioxidants in the human body. Antioxidant Nutraceuticals.

[45] Cruz VA, de Albuquerque CP, Guimarães MF (2023). New insights at the interface between leprosy and immune-mediated rheumatic diseases. Front Med (Lausanne).

[46] Chauhan M, Sharma P, Sharma L (2021). Oxidative stress in borderline and lepromatous leprosy. Indian J Lepr.

[47] Sharma N, Mahajan V (2023). Differential diagnosis of dermatological disorders in relation to leprosy. IAL Textbook of Leprosy.

[48] Lasry-Levy E, Hietaharju A, Pai V, Ganapati R, Rice AS, Haanpää M, Lockwood DN (2011). Neuropathic pain and psychological morbidity in patients with treated leprosy: a cross-sectional prevalence study in Mumbai. PLoS Negl Trop Dis.

[49] Haroun OM, Hietaharju A, Bizuneh E (2012). Investigation of neuropathic pain in treated leprosy patients in Ethiopia: a cross-sectional study. Pain.

[50] Raicher I, Stump PR, Baccarelli R (2016). Neuropathic pain in leprosy. Clin Dermatol.

[51] Khadilkar SV, Patil SB, Shetty VP (2021). Neuropathies of leprosy. J Neurol Sci.

[52] Rao PN, Suneetha S (2016). Pure neuritic leprosy: current status and relevance. Indian J Dermatol Venereol Leprol.

[53] Rodrigues Júnior IA, Gresta LT, Noviello Mde L, Cartelle CT, Lyon S, Arantes RM (2016). Leprosy classification methods: a comparative study in a referral center in Brazil. Int J Infect Dis.

[54] Alves PH, Cirino FO, Garcia LP (2024). Median nerve impairment in leprosy: how does it differ from the classic carpal tunnel syndrome?. Arq Neuropsiquiatr.

[55] Ooi WW, Srinivasan J (2004). Leprosy and the peripheral nervous system: basic and clinical aspects. Muscle Nerve.

[56] Reja AH, Biswas N, Biswas S (2013). Fite-Faraco staining in combination with multiplex polymerase chain reaction: a new approach to leprosy diagnosis. Indian J Dermatol Venereol Leprol.

[57] Lugão HB, Frade MA, Marques W Jr, Foss NT, Nogueira-Barbosa MH (2016). Ultrasonography of leprosy neuropathy: a longitudinal prospective study. PLoS Negl Trop Dis.

[58] Chen X, Di L, Qian M, Shen D, Feng X, Zhang X (2024). Neurological features of Hansen disease: a retrospective, multicenter cohort study. Sci Rep.

[59] Jindal R, Shirazi N (2016). Uncommon clinical presentations of leprosy: apropos of three cases. Lepr Rev.

[60] Kabir H, Hossain S (2019). Knowledge on leprosy and its management among primary healthcare providers in two districts of Bangladesh. BMC Health Serv Res.

[61] Grijsen ML, Nguyen TH, Pinheiro RO, Singh P, Lambert SM, Walker SL, Geluk A (2024). Leprosy. Nat Rev Dis Primers.

[62] Abe M, Ozawa T, Minagawa F, Yoshino Y (1981). Subclinical infection in leprosy‐‐its detection and control by fluorescent leprosy antibody absorption (FLA‐ABS) test. Lepr Rev.

[63] Sivakumaran P, Barros B, Antonio Dias VL, Lockwood DN, Walker SL (2024). A retrospective cohort study of monthly rifampicin, ofloxacin and minocycline in the management of leprosy at the Hospital for Tropical Diseases, London, United Kingdom. PLoS Negl Trop Dis.

[64] Banstola NL, Hasker E, Mieras L (2024). Effectiveness of ongoing single dose rifampicin post-exposure prophylaxis (SDR-PEP) implementation under routine programme conditions-an observational study in Nepal. PLoS Negl Trop Dis.

[65] Lau KH (2019). Neurological complications of leprosy. Semin Neurol.

[66] Pierneef L, van Hooij A, de Jong D (2024). Rapid test for Mycobacterium leprae infection: a practical tool for leprosy. Infect Dis Poverty.

[67] Brito Gonçalves BE, Raiol AM, Brito AV, Silva MJ, Sardinha DM, Lima KV, Lima LN (2024). Prevalence of paucibacillary cases of leprosy in Brazil: a 20-year systematic review and meta-analysis. Front Med (Lausanne).

[68] Smith CS, Aerts A, Saunderson P, Kawuma J, Kita E, Virmond M (2017). Multidrug therapy for leprosy: a game changer on the path to elimination. Lancet Infect Dis.

[69] Van Brakel WH, Anderson AM, Withington SG (2003). The prognostic importance of detecting mild sensory impairment in leprosy: a randomized controlled trial (TRIPOD 2). Lepr Rev.

[70] Richardus JH, Withington SG, Anderson AM, Croft RP, Nicholls PG, Van Brakel WH, Smith WC (2003). Treatment with corticosteroids of long-standing nerve function impairment in leprosy: a randomized controlled trial (TRIPOD 3). Lepr Rev.

[71] Marlowe SN, Hawksworth RA, Butlin CR, Nicholls PG, Lockwood DN (2004). Clinical outcomes in a randomized controlled study comparing azathioprine and prednisolone versus prednisolone alone in the treatment of severe leprosy type 1 reactions in Nepal. Trans R Soc Trop Med Hyg.

[72] Rao PS, Sugamaran DS, Richard J, Smith WC (2006). Multi-centre, double blind, randomized trial of three steroid regimens in the treatment of type-1 reactions in leprosy. Lepr Rev.

[73] Garbino JA, Virmond Mda C, Ura S, Salgado MH, Naafs B (2008). A randomized clinical trial of oral steroids for ulnar neuropathy in type 1 and type 2 leprosy reactions. Arq Neuropsiquiatr.

[74] Walker SL, Nicholls PG, Dhakal S (2011). A phase two randomised controlled double blind trial of high dose intravenous methylprednisolone and oral prednisolone versus intravenous normal saline and oral prednisolone in individuals with leprosy type 1 reactions and/or nerve function impairment. PLoS Negl Trop Dis.

[75] Furness M (1982). Physiotherapy in leprosy. Aust J Physiother.

[76] Álvarez CCS, Hans Filho G (2019). Leprosy and physiotherapy: a necessary approach. J Hum Growth Dev.

[77] Kaada B, Emru M (1988). Promoted healing of leprous ulcers by transcutaneous nerve stimulation. Acupunct Electrother Res.

[78] Abdul Rahman N, Rajaratnam V, Burchell GL, Peters RM, Zweekhorst MB (2022). Experiences of living with leprosy: a systematic review and qualitative evidence synthesis. PLoS Negl Trop Dis.

[79] Mohammed AK, Lalonde DH (2019). Wide awake tendon transfers in leprosy patients in India. Hand Clin.

[80] Reyila VP, Betsy A, Riyaz N, Sasidharanpillai S, Sherjeena PV, Majitha MP, Joseph DM (2019). Clinico-epidemiological study of disability due to leprosy at the time of diagnosis among patients attending a tertiary care institution. Indian J Dermatol.

[81] Miyashiro D, Cardona C, Valente NY, Avancini J, Benard G, Trindade MA (2019). Ulcers in leprosy patients, an unrecognized clinical manifestation: a report of 8 cases. BMC Infect Dis.

[82] Costa RM, Fernandes MA, Zagonel IP (2024). Transitions experienced by people living with limitations resulting from leprosy: a research-care study. Rev Bras Enferm.

[83] Laura Gillini, Erwin Cooreman, Basudev Pandey (2018). Implementing the Global Leprosy Strategy 2016-2020 in Nepal: lessons learnt from active case detection campaigns. Lepr Rev.

[84] Goulart IM, Goulart LR (2008). Leprosy: diagnostic and control challenges for a worldwide disease. Arch Dermatol Res.

[85] Lockwood DN (2019). Chronic aspects of leprosy-neglected but important. Trans R Soc Trop Med Hyg.

[86] Cross H (2006). Interventions to address the stigma associated with leprosy: a perspective on the issues. Psychol Health Med.

[87] Rafferty J (2005). Curing the stigma of leprosy. Lepr Rev.

[88] Sermrittirong S, Van Brakel WH (2014). Stigma in leprosy: concepts, causes and determinants. Lepr Rev.

[89] Putri AI, de Sabbata K, Agusni RI (2022). Understanding leprosy reactions and the impact on the lives of people affected: an exploration in two leprosy endemic countries. PLoS Negl Trop Dis.

[90] Jain S, Visser LH, Suneetha S (2016). Imaging techniques in leprosy clinics. Clin Dermatol.

[91] Das M, David D, Horo I, Van Hooij A, Tió-Coma M, Geluk A, Vedithi SC (2023). Mycobacterium leprae and host immune transcriptomic signatures for reactional states in leprosy. Front Microbiol.

[92] Barbieri RR, Xu Y, Setian L (2022). Reimagining leprosy elimination with AI analysis of a combination of skin lesion images with demographic and clinical data. Lancet Reg Health Am.

[93] Lopes-Luz L, Saavedra DP, Fogaça MB, Bührer-Sékula S, Stefani MM (2023). Challenges and advances in serological and molecular tests to aid leprosy diagnosis. Exp Biol Med (Maywood).

[94] Yang D, Shui T, Miranda JW (2016). Mycobacterium leprae-infected macrophages preferentially primed regulatory t cell responses and was associated with lepromatous leprosy. PLoS Negl Trop Dis.

[95] Graham L Jr, Navalkar RG (1984). Evaluation of Mycobacterium leprae immunogenicity via adoptive transfer studies. Infect Immun.

[96] Moraes MO, Sarno EN, Teles RM, Almeida AS, Saraiva BC, Nery JA, Sampaio EP (2000). Anti-inflammatory drugs block cytokine mRNA accumulation in the skin and improve the clinical condition of reactional leprosy patients. J Invest Dermatol.

[97] Liem G The leprosy project nutrition program guideline and workbook. Dedicated to all malnourished children of the people affected by leprosy. http://www.theleprosyproject.org/uploads/1/4/3/8/14387444/nutrition_program_guideline_and_workbook.pdf.

[98] Dasgupta S, Mukherjee S, Bagchi C (2024). Efficacy of leprosy vaccines across the globe: a systematic review & meta-analysis of randomized controlled trials. Indian J Med Res.

